# Surgical technique of low-profile dual plating for midshaft clavicle fractures

**DOI:** 10.1007/s00064-025-00903-y

**Published:** 2025-06-13

**Authors:** Bryan J. M. van de Wall, Nadine Diwersi, Lukas Scheuble, Yannic Lecoultre, Björn Christian Link, Reto Babst, Frank J. P. Beeres

**Affiliations:** 1 Department of Orthopedics and Traumatology, Luzerner Kantonsspital, Spitalstraße, 6000 Luzern 16, Switzerland; 2 Department Health Sciences and Medicine, University of Luzern, Luzern, Switzerland; 3 Department of Surgery, Kantonsspital Obwalden, Sarnen, Switzerland

**Keywords:** Bone fractures, Fracture fixation, Prostheses and implants, Meta-analysis, Biomechanical properties, Knochenfrakturen, Frakturstabilisierung, Prothesen und Implantate, Metaanalyse, Biomechanische Eigenschaften

## Abstract

**Objective:**

The aim of this surgical technique is fracture healing with anatomical alignment and less implant irritation due to smaller, low-profile plates. Equal to superior stability is provided compared to single superior- or anterior-based plates.

**Indications:**

The same general indications for surgical stabilization of clavicle fractures apply for low-profile double plating and include fracture displacement of one or more shaft width, shortening of more than 1 cm in length, and patients with high physical activity levels. Double plating is especially suitable for fractures in the midportion of the clavicle.

**Contraindications:**

Fractures in the far lateral portion of the clavicle due to physiological thinning of the clavicle potentially causing problems with screw purchase of screws fitted in the anterior plate.

**Surgical technique:**

A 2.0 mm low-profile mini plate is used on the superior and a 2.4 or 2.7 mm on the anterior surface of the clavicle. The plates are fixated with a minimum of two cortical or locking screws on each side of the fracture in each plate. A lag screw can be used if absolute stability can be obtained in simple fractures.

**Postoperative management:**

A standard functional postoperative regime can be followed after plate fixation with free mobilization up to 90° without weight bearing for 6 weeks. Afterwards free range of motion and weight bearing are allowed.

**Results:**

A biomechanical study, meta-analysis, and retrospective analysis have shown that low profile double plating offers equal to superior stability, lower rates of implant irritation and subsequent removal compared to conventional single plating with equal healing potential.

## Abstract (de)

**Zielsetzung:**

Ziel dieser Operationstechnik ist die Frakturheilung mit anatomischer Ausrichtung und geringerer Implantatirritation durch kleinere, niedrigprofilige Platten. Damit wird eine im Vergleich zu einzelnen superioren oder anterioren Platten gleichwertige bis bessere Stabilität erreicht.

**Indikationen:**

Für die Low-Profile-Doppelverplattung gelten die gleichen allgemeinen Indikationen für die chirurgische Stabilisierung von Klavikulafrakturen wie für die Verschiebung der Fraktur um eine oder mehrere Schaftbreiten, Verkürzungen von mehr als 1 cm Länge und Patienten mit hoher körperlicher Aktivität. Die Doppelverplattung eignet sich besonders für Frakturen im mittleren Bereich des Schlüsselbeins.

**Kontraindikationen:**

Frakturen im weit seitlichen Teil der Klavikula aufgrund der physiologischen Ausdünnung, die Probleme beim Schraubeneinzug der in die anteriore Platte eingesetzten Schrauben verursachen kann.

**Chirurgische Technik:**

Eine 2,0-mm-Miniplatte mit niedrigem Profil wird auf der oberen, eine 2,4- oder 2,7-mm-Platte auf der vorderen Oberfläche der Klavikula verwendet. Die Platten werden mit mindestens 2 Kortikalis- oder Verriegelungsschrauben auf jeder Seite der Fraktur in jeder Platte fixiert. Eine Zugschraube kann verwendet werden, um bei einfachen Frakturen absolute Stabilität zu erreichen.

**Postoperatives Management:**

Nach der Plattenfixation kann ein standardmäßiges postoperatives Funktionsregime mit freier Mobilisierung bis zu 90° ohne Gewichtsbelastung für 6 Wochen durchgeführt werden. Danach sind freie Beweglichkeit und Gewichtsbelastung erlaubt.

**Ergebnisse:**

Eine biomechanische Studie, eine Metaanalyse und eine retrospektive Analyse haben gezeigt, dass die Low-Profile-Doppelverplattung im Vergleich zur konventionellen Einzelverplattung bei gleichem Heilungspotenzial eine gleichwertige bis überlegene Stabilität, geringere Raten von Implantatirritationen und eine spätere Entfernung bietet.

## Introductory remarks

Accounting for 2–5% of all fractures in young adults, clavicle fractures are common injuries in this age category [1]. They can be classified according to the AO/OTA (Arbeitsgemeinschaft für Osteosynthesefragen/Orthopaedic Trauma Association) classification. Surgical treatment is generally advised in cases with more than one shaft width displacement or a shortening of more than 1 cm [1]. There are several surgical treatment options for clavicle fractures such as open or minimally invasive anterior and superior plate fixation or intramedullary nailing [2, 3]. A major disadvantage of plate fixation, however, is implant irritation. As a result, up to 64% of patients undergo implant removal after healing is attained [4]. With rising costs and an increasing burden on the healthcare system, it is vital to find solutions to reduce the risk of irritation and the associated need for secondary removal [5].

Low profile dual plate fixation has recently emerged as a new promising technique [6]. Using the same approach to the fracture zone as in traditional open plate fixation, dual plate fixation uses two smaller plates (2.7 mm or smaller) positioned on the anterior and superior aspect of the clavicle to stabilize the fracture, instead of using a 3.5 mm or newer generation 2.7 mm single plate.

Due to the plates’ smaller size and lower profile, the double plate technique is believed to cause less implant irritation and, consequently, a lower need for removal [7]. Biomechanical studies suggest equal to superior stability, especially on multidimensional biomechanical stress tests and torsional rigidity, compared to superior- or anterior-based plates [8–10]. As an additional advantage, less postoperative numbness is to be expected due to smaller incisions needed to seat the smaller plates on the surface of the clavicle.

The goal of the current paper is to provide a description of the surgical technique for low-profile double plating and present our clinical experience with it.

## Surgical principles and objective

The main goals of low-profile dual plating of midshaft clavicle fractures are to offer a safe procedure with high union rates, equal to higher biomechanical stability, and less implant prominence potentially leading to removal of fewer implants.

## Advantages

- High union rate
- Potentially less implant irritation and the need for removal
- Better cosmetic outcome due to smaller incision
- Lower costs of implants
- Less numbness due to smaller incision

## Disadvantages

- More surgical dissection to seat two plates on the clavicle potentially negatively impacting blood supply, and thus healing and risk of infection of fracture site(s)
- Insufficient stability leading to implant failure if basic principles of fracture fixation are not followed
- Potentially more difficult implant removal due to ingrown implant

## Indications

The same general indications for surgical stabilization of clavicle fractures apply for low-profile double plating and include

- Fracture displacement of one or more shaft width
- Shortening of more than 1 cm in length
- Patient with high physical activity level

Low profile double plating is especially suitable for

- Midshaft clavicle fractures
- Both simple (AO/OTA 15.2A and Robinson 2A), wedge (AO/OTA 15.2B and Robinson 2B), and comminuted fractures (AO/OTA 15.2C and Robinson 2C) can be stabilized using the double plating technique.

## Contraindications

Contraindications specifically for low-profile double plating include the following:

- Fractures in the lateral portion of the clavicle. The lateral part of the clavicle becomes increasingly thinner potentially causing problems with screw purchase of screws inserted in the anterior plate.

## Preoperative work up

- Assessment of medical history and physical exam including
  - Age, comorbidities, medication, allergies
  - Assessment of occupational demands
  - Assessment of neurovascular status
  - Assessment of skin lesions from accident or pre-existing scars
- Radiographs of clavicle (anteroposterior and tangential views)
- Proper planning of surgery including sketches, etc.
- Antibiotic prophylaxis (30–45 min prior to incision)

## Instruments and implants

- Radiolucent operating table with head holder
- Low-profile 2.0 mm plate for superior positioning and 2.4 or 2.7 mm low-profile plate for anterior side
- Corresponding locking and nonlocking screws
- Intraoperative x‑ray
- Standard surgical instruments for osteosynthesis and soft tissue protection

## Anesthesia and positioning

- General endotracheal anesthesia
- Supine positioning on a radiolucent table with slightly elevated back and small sandbag or pillow between scapulae
- Placement of image intensifier at the cranial side of and parallel to the patient to ensure visualization of the entire clavicle in anteroposterior and caudocranial views

## Surgical technique

**Fig. 1:**
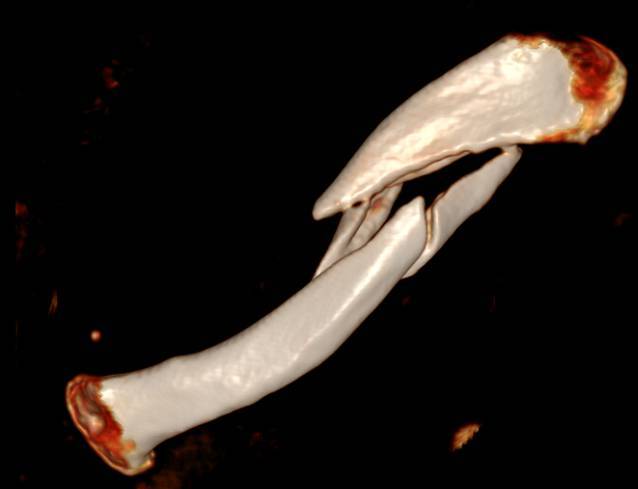
Preoperative three-dimensional reconstruction of a left clavicle midshaft fracture in a 67-year-old man

**Fig. 2:**
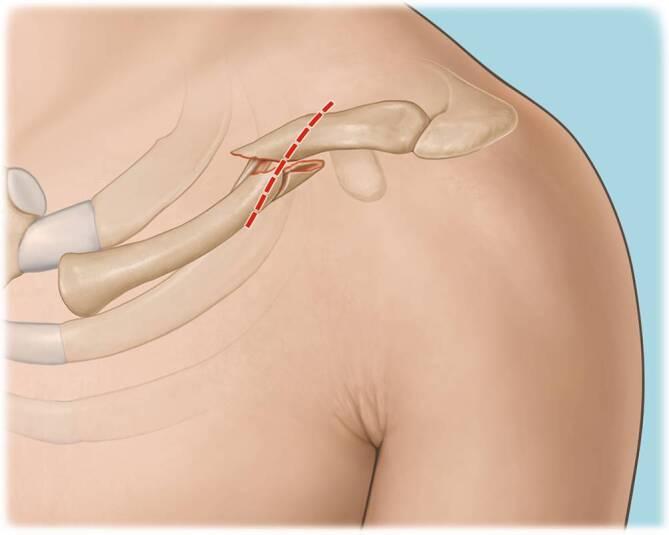
Drawing of anatomical landmarks including skin incision. An incision in the skin lines is preferred for better cosmetic results

**Fig. 3:**
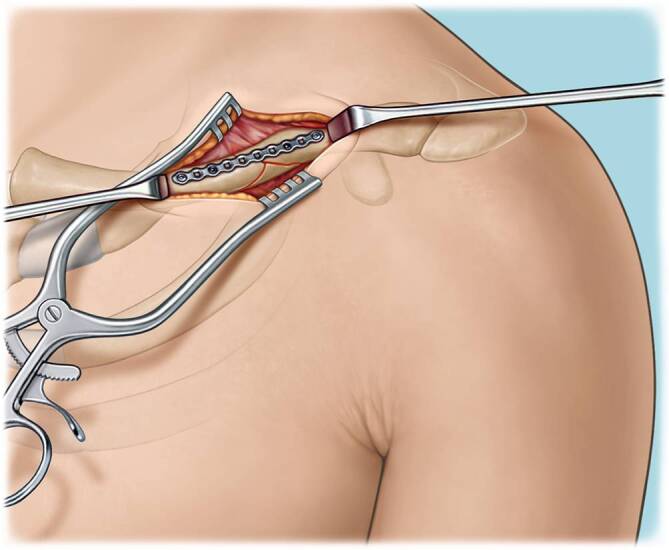
The fracture site is cleaned and the fracture is reduced using reduction forceps, k‑wires or lag screws depending on the preference of the surgeon. A 2.0 mm plate is positioned on the superior surface of the clavicle. It is suggested to use a plate that has a minimum of 3 holes on each side of the fracture. In good bone quality, one cortical screw on each side of the fracture may be used to pull the plate onto the bone. In the remaining plate holes, locking screws are preferably used. A minimum of two screws on each side of the fracture is required. In case of poor bone quality, consider prebending the plate and using three locking screws on each side of the fracture. The 2 mm plate should provide enough stability to hold the reduction. Removal of the reduction tools should be possible at this stage

**Fig. 4:**
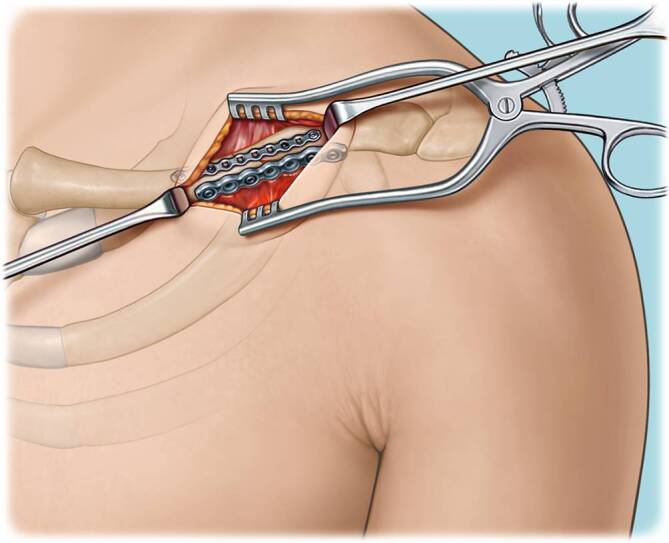
A second 2.7 mm is prepared. It is important to prebend the plate as the use of locking screws in this plate is advisable for adequate stability based on biomechanical studies explained in more detail in the results section. Care should be taken not to compromise the locking holes when bending the plate by bending in between the holes. A minimum of two locking screws on each side of the fracture is required. Depending on fracture morphology and bone quality, three screws on each side may be considered. The 2.7 mm plate is seated on the anterior surface of the clavicle. K‑wires may be used in combination with a reduction forceps to hold the plate in the correct position and compress the plate onto the anterior surface of the clavicle. In this case a total of five locking screws were placed in the anterior plate

**Fig. 5:**
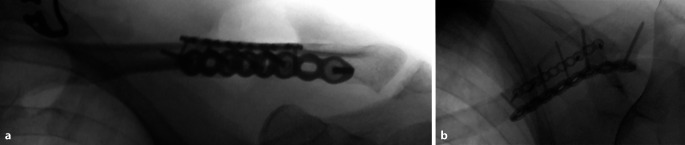
Intraoperative fluoroscopy (images taken in two directions) is used to assess reduction, implant positioning, and screw length. The platysma is closed with 3.0 soluble sutures and the skin with a running suture with 4.0 thread. **a** Anteroposterior view osteosynthesis, **b** Axial view osteosynthesis

(Figs. 1, 2, 3, 4 and 5)

## Postoperative management

- Wound dressing with simple patch
- Routine wound control according to local protocol
- Removal of stitches after 12–14 days
- Standard local guidelines on limitations with regard to the range of motion and weightbearing for single plating can also be used for patients treated with low profile double plates. In the author’s hospital, patients are allowed to actively elevate their arm to 90° abduction/anteflexion. Weightbearing is not allowed in the first 6 weeks. After this period, patients are allowed to freely move their arm and weightbearing as tolerated.
- If needed, a sling for analgesic reasons can be advocated for the first couple of days
- Conventional radiographs after 6 weeks, 3 months (and 1 year)
- The implants are not routinely removed unless at patients’ request. Generally, a minimal interval of 12 months between osteosynthesis and removal of both plates should be applied to reduce the refracture risk to 8% [11]. Earlier removal carries a 28% refracture risk.

## Errors, hazards, complications

- Iatrogenic injury to the neurovascular bundle requiring open or endovascular repair.
- Iatrogenic perforation of the thoracic cavity with subsequent (tension) pneumothorax requiring placement of a thoracic tube.
- Fracture-related infections necessitating debridement and potentially implant replacement with antibiotic treatment for 3 months.
- Malunion and non-union. Depending on whether patients are symptomatic, revision surgery can be performed.
- Implant irritation requiring surgical removal of the plates and screws.

## Results

Essentially, the idea of low-profile double plating is to provide the same stability and healing potential as conventional single plating, while simultaneously reducing the high implant irritation rate in the clavicular region that has plagued surgery. Our research group has performed multiple studies on this topic showing promising results; however, certain aspects need to be acknowledged.

A biomechanical study was performed comparing different low profile double plating configurations (2 mm superior/2 mm anterior and 2 mm superior/2.5 mm anterior, Synthes matrix mandible plates [DePuy Synthes, Raynham, MA, USA]) to a single 3.5 mm superior plate in AO/OTA 15.2C fractures [9]. The study revealed that the 2.0/2.0 mm dual plates had similar initial stiffness, but significantly lower resistance to failure compared to single 3.5 mm plates. The 2.5/2.0 configuration demonstrated considerably higher initial stiffness and similar resistance to failure. Notably, three locking screws were used on each side of the fracture in each plate. Although it remains difficult to translate these results into clinical implications, it may be concluded that the 2.5/2.0 mm configuration is a safe alternative to conventional plating. Three other biomechanical studies found comparable results, be it with different plate type, dimensions and fracture type (Table 1). These studies formed the basis for advising the use of a 2.0 mm superiorly and a 2.4 mm or 2.7 mm plate anteriorly (depending on size of the patient). It should be acknowledged that the effect of number and type of screws per plate has not been thoroughly investigated yet. Therefore, it remains unclear what the minimum requirements are in this particular field to attain the same biomechanical stability as conventional single plating.

**Table 1:** Fracture characteristics and implants used in biomechanical studies that found similar (or improved) stability of double plating compared with single plates

Author	Year	Manufacturer	Double plates		Screws per plate	AO/OTA fracture type
			Superior (mm)	Anterior (mm)		
Ziegler [14]	2019	Acumed, Hillsboro, OR, USA	2.7	2.7	4 cortical	B
Kitzen [15]	2022	Synthes, DePuy, Raynham, MA, USA	2.4	2.7	4 cortical	B
Ferguson [16]	2023	Stryker, Portage, MI, USA	2.3	2.3	1 locking/2 cortical	A

A meta-analysis also showed favorable results for low profile double plating [7]. Four studies were included that compared 3.5 mm single plating to different configurations and combinations of double plates ranging from 2.0 to 2.7 mm plates (Table 2). It was found that both single and double plating yield the same healing rates (99% versus 98%, respectively). However, re-intervention was significantly more often performed in the single plating group (16% versus 6%) mainly caused by higher rates of implant irritation (12% versus 5%). Subgroup analysis on the effect of number and type of screws was not possible as this was not reported in the included studies.

**Table 2:** Plates used for double plating in studies included in the meta-analysis

Author	Year	Double plating implants
DeBaun [17]	2020	LCP anterior and superior 2.7, 2.4, or 2.0 mm
Allis [18]	2020	LCP 2.7 mm superior and 2.4 mm anterior
Lee [19]	2019	LCP anterior and superior 2.7 mm plates
Chen [20]	2016	LCP 2.7 mm and aid plate (not further specified)

Between 2020 and 2022, 99 patients were treated for midshaft clavicle fractures at our level one trauma center: 74 with single and 25 with double plating (Table 3). All operation were performed by or under direct supervision of a (senior) consultant orthopedic trauma surgeon. In almost all patients in the double plating group, a combination of a 2 mm plate superior and 2.7 mm plate anterior was used. The median length of both superior and anterior plates was 8 (IQR 3) holes. A median number of 2 screws was used on each side of the fracture per plate. Mostly cortical screws were used in the superior and locking screws in the anterior plate. Also in our study population, the need for implant removal for irritation was significantly lower in the double plating group (20% versus 48%) compared to single plating. As an additional finding, the operation duration (measured from incision to closure) was shorter for double plating (median 95 min versus 110 min). Given the fact that patient and fracture characteristics were comparable in both treatment groups, a potential explanation could be that patients in the double plating group were operated on by a more experienced surgeon (senior consultant 24% versus 15%). Notably, the difference in level of expertise was not statistically significant. How much the surgical technique contributed to the shorter surgical duration remains unclear. Despite one patient in the double plating group who had an implant failure because of a fall on the shoulder (plates were bent on X‑ray; however, patient was asymptomatic), all fractures healed in both groups.

**Table 3:** Baseline characteristics single and double plating group retrospective cohort study

	Single plating	Double plating	Difference
	(*n* = 74)	(*n* = 25)	*p*
Males (%)	61 (82.4%)	18 (72.0%)	0.261
Age in years (median, IQR)	46 (25)	46 (29)	0.646
ASA score (median, IQR)	2 (1)	2 (3)	0.657
HET (*n*, %)	23 (31.1%)	8 (32.0%)	0.932
ISS ≥ 16 (*n*, %)	15 (20.2%)	8 (32.0%)	0.230
Ipsilateral injury (*n*, %)	23 (31.1%)	9 (36.0%)	0.649
Fracture type (*n*, %)			
*Simple*	25 (33.8%)	11 (44.0%)	0.656
*Wedge*	7 (9.5%)	2 (8.0%)	
*Multifragmentary*	42 (56.8%)	12 (48.0%)	

*n* number, *%* is percentage of total population, *SD *standard deviation, *IQR* interquartile range, *ASA* American Society of Anaesthesiologists, *HET* High Energy Trauma, *ISS* Injury Severity Score

It is important to note that there is considerable heterogeneity within and between studies regarding plate dimensions and type and number of screws used for double plating. This stems from the fact that it is a relatively new technique and surgeons are still exploring its possibilities causing these differences. Presented results, therefore, solely give an impression what may be expected from double plating in general. The optimal implant dimensions have yet to be defined.

There remain some concerns regarding an increased risk of refracture after removal of the two plates compared to removal of a single plate. Although clinical studies are lacking, a biomechanical study showed that there was no difference in the amount of force required for refractures to occur when single plating was compared to double plating [12].

All in all, results for low profile double plating are promising. Currently, a prospective natural experiment study is being conducted at our clinic aiming to provide higher quality evidence also allowing analyses on optimal plate and screw configuration and dimensions [13]. To date, half of the required 336 patients have been included and results are expected in 2026.

## Data Availability

The data that support the findings of this study are available from the corresponding author upon reasonable request.

## Declarations

For this article no studies with human participants or animals were performed by any of the authors. All studies mentioned were in accordance with the ethical standards indicated in each case.
